# Local adaptation through countergradient selection in northern populations of *Skeletonema marinoi*

**DOI:** 10.1111/eva.13436

**Published:** 2022-07-11

**Authors:** Josefin Sefbom, Anke Kremp, Per Juel Hansen, Kerstin Johannesson, Anna Godhe, Karin Rengefors

**Affiliations:** ^1^ Department of Marine Sciences University of Gothenburg Gothenburg Sweden; ^2^ Marine Research Centre Finnish Environment Institute (SYKE) Helsinki Finland; ^3^ Biological Oceanography Leibniz Institute for Baltic Sea Research Warnemünde Rostock Germany; ^4^ Marine Biological Section University of Copenhagen Helsingør Denmark; ^5^ Department of Marine Sciences – Tjärnö University of Gothenburg Strömstad Sweden; ^6^ Aquatic Ecology, Department of Biology Lund University Lund Sweden

**Keywords:** countergradient variation, diatom, intraspecific competition, local adaptation, pH, phenotypic plasticity, salinity

## Abstract

Marine microorganisms have the potential to disperse widely with few obvious barriers to gene flow. However, among microalgae, several studies have demonstrated that species can be highly genetically structured with limited gene flow among populations, despite hydrographic connectivity. Ecological differentiation and local adaptation have been suggested as drivers of such population structure. Here we tested whether multiple strains from two genetically distinct Baltic Sea populations of the diatom *Skeletonema marinoi* showed evidence of local adaptation to their local environments: the estuarine Bothnian Sea and the marine Kattegat Sea. We performed reciprocal transplants of multiple strains between culture media based on water from the respective environments, and we also allowed competition between strains of estuarine and marine origin in both salinities. When grown alone, both marine and estuarine strains performed best in the high‐salinity environment, and estuarine strains always grew faster than marine strains. This result suggests local adaptation through countergradient selection, that is, genetic effects counteract environmental effects. However, the higher growth rate of the estuarine strains appears to have a cost in the marine environment and when strains were allowed to compete, marine strains performed better than estuarine strains in the marine environment. Thus, other traits are likely to also affect fitness. We provide evidence that tolerance to pH could be involved and that estuarine strains that are adapted to a more fluctuating pH continue growing at higher pH than marine strains.

## 1 INTRODUCTION

Landscape and seascape heterogeneity provide a variety of habitats to which populations of species might either specialize by local adaptation (Kawecki & Ebert, 2004) or tolerate by being phenotypically plastic (Bradshaw, 1965; Pelletier et al., 2009). In the former case, phenotypes poorly suited to the local habitat are removed by selection, and the fitness of the local population is tweaked towards a local trait optimum, however, balanced by an inflow of maladaptive alleles (resulting in a ‘selection‐migration’ balance). Alternatively, phenotypic plasticity allows for the spread of general and plastic genotypes over multiple habitats and will prevent local adaptation in the absence of selection. Plasticity is expected in temporally heterogeneous and/or fine‐grained environments (sensu Levins, 1968) and when cost is low (Pigliucci, 2001). Together this highlights the complex interplay between gene flow, local adaptation and also plasticity (Crispo, 2008; Räsänen & Hendry, 2008).

The rate and scale at which gene flow can occur are dependent on a species' ability for dispersal (movement of individuals; Bohonak, 1999) and colonization, that is, survival and reproduction in the new environment (Svensson et al., 2017). Microalgae fall into the size fraction (<1 mm) of organisms that have been suggested to have an unlimited dispersal potential (Finlay, 2002). Despite their assumed ubiquitous dispersal, research during the past decade has shown that gene flow in most of the investigated microalgae is limited and that populations are typically genetically differentiated (Logares, Boltovskoy, et al., 2009; Medlin, 2007; Vanormelingen et al., 2008). At the same time, there are studies showing very low levels of genetic differentiation of marine phytoplankton (e.g. Evans et al., 2005).

The question as to whether microalgal populations are locally adapted or rely on phenotypic plasticity remains largely unanswered. Evidence from physiological studies on genetically differentiated microalgae populations has revealed that populations can exhibit distinct optima for growth (Kremp et al., 2012; Rynearson & Armbrust, 2004; Sjöqvist et al., 2015; Verma et al., 2020), suggestive of adaptive genetic divergence. Similarly, Rengefors et al. (2015) showed high plasticity in salinity tolerance in a dinoflagellate species, yet with the growth optima skewed towards local conditions, suggesting local adaptation. Other studies show high phenotypic diversity within populations yet no local adaptation (Sassenhagen, Wilken, et al., 2015). The very large population sizes of microalgal populations (10^4^ to 10^7^ individuals per L water) generating a high genetic diversity, together with short generation time (mitotic cell division), will facilitate rapid evolution and hence local adaptation. This is similar to what has been observed in plants (Leimu & Fisher, 2008) and fish (Barrio et al., 2016) with large populations and modelled for in phytoplankton (Lynch et al., 1991). To date, only a limited number of studies have specifically addressed the role of local adaptation in microalgal population differentiation and these provide evidence for both plasticity (Rengefors et al., 2015; Sassenhagen, Wilken, et al., 2015) and local adaptation (Boenigk et al., 2007; O'Donnell et al., 2018; Sjöqvist et al., 2015; Weisse et al., 2011; Yvon‐Durocher et al., 2017).

Adaptive differentiation has also been suggested to be the cause of reduced gene flow between populations (Nosil, 2008; Nosil et al., 2009; Orsini et al., 2013; Räsänen & Hendry, 2008). Establishment success of immigrants can then be greatly reduced because they will be outcompeted by individuals from the locally adapted population, and thus local adaptation becomes a driver of population differentiation (e.g. De Meester et al., 2016). In those cases, it is not the suboptimal environmental conditions per se that hinder establishment success, because in the absence of competition immigrants may still be able to sustain population stability long enough to also become locally adapted. Considering the potential for large‐scale dispersal in microalgae, competitive exclusion achieved through local adaptation may provide a potential mechanism for driving population genetic differentiation exhibited over small‐spatial scales. Studies focused on population genetic structure in fact suggest that patterns of highly differentiated populations could be a result of competitive exclusion of new immigrants (Rengefors et al., 2012; Sassenhagen, Sefbom, et al., 2015; Sefbom et al., 2015; Sildever et al., 2016), aka the Monopolization hypothesis (De Meester et al., 2002, 2016).

*Skeletonema marinoi* (Bacillariophyceae) is a centric marine diatom common during the spring bloom in many coastal temperate regions (Kooistra et al., 2008). Despite the wide geographic range of *S. marinoi*, this species displays population genetic differentiation in putatively neutral markers (microsatellites) even over small spatial scales (<7 km; Sefbom et al., 2018). Although *Skeletonema* is an ancestrally marine genus, it has also successfully established in estuarine and brackish water habitats, such as the Baltic Sea. Due to permanently low‐saline conditions, the Baltic Sea exhibits very low species diversity and is considered an ecologically marginal marine ecosystem (Johannesson & André, 2006). In this sea, there is a stable salinity gradient, ranging from 10 to 12 PSU in the Danish straits (at the entrance in the south) and gradually decreasing northward, to almost freshwater conditions in the northern Baltic Sea (<2 PSU). Permanently low salinities in the central and northern parts of the Baltic Sea are linked to low alkalinity and decreased buffering capacity (Müller et al., 2016), potentially increasing vulnerability of the organisms living here to pH fluctuations. *S. marinoi* has proven to be highly euryhaline with strains being able to sustain growth at salinity conditions ranging from 7 to 33 PSU (Balzano et al., 2011). As a result, *S. marinoi* is found as far north as in the Bothnian Sea, where salinity ranges between 4 and 6 PSU (Sjöqvist et al., 2015).

In general, the transition between different salinity conditions is as a major obstacle for aquatic organisms to overcome (Lee & Bell, 1999), and variation in salinity seems to be a strong selective force for a number of marine multicellular species at the Baltic Sea entrance (Johannesson et al., 2020). Also in microalgae, salinity appears to play an important role in species divergence (Logares, Schalchian‐Tabrizi, et al., 2009). Recently, it was suggested that the population genetic differentiation displayed between populations of *S. marinoi* inside and outside the Baltic Sea could be explained by local adaptation to different salinities (Sjöqvist et al., 2015). Moreover, Godhe et al. (2016) showed that the open water population of *S. marinoi* in the Baltic sea was significantly differentiated across a transect including both a salinity gradient and a geographic distance. A recent study by Pinseel et al. (2022) reported profound responses of Baltic *S. marinoi* strains to salinity changes at the level of gene expression, specifically regarding cellular metabolism, nutrient demand and stress response. The responses included upregulation of biosynthesis of storage compounds, carbon fixation as well as upregulation of genes related to nutrient uptake and oxidative stress. Although the latter did not examine the expression in relation to strain origin, it suggests that salinity is likely an important selective pressure in *S. marinoi*.

The purpose of this study was to experimentally determine whether local adaptation could contribute significantly to genetic differentiation of marine microalgal populations, which theoretically should experience high gene flow. Specifically, we tested for evidence of local adaptation of strains of *S. marinoi* from two genetically differentiated populations (*F*_ST_ 0.07–0.09; Sjöqvist et al., 2015), originating from a marine (Kattegat‐Skagerrak) and an estuarine (Bothnian Sea) environment, respectively. Maximum growth rates were used as indicators of fitness (Belotte et al., 2003; Gross et al., 2018). Our hypothesis was that local genotypes would grow faster and/or produce more biomass in their native water than in non‐native water. Furthermore, we expected that this would lead to a competitive advantage for the local over the foreign strain in the local environment. Finally we monitored pH in relation to strain growth since both salinity and buffering capacity vary between marine and estuarine water, where estuarine waters have lower buffering capacity than marine water (Key et al., 2004; Thomas & Schneider, 1999).

## 2 MATERIAL AND METHODS

### 2.1 *Skeletonema marinoi* strains

We used a total of nine nonaxenic clonal isolates originating from the Bothnian Sea, northern Baltic Sea (Sjöqvist et al., 2015), and six nonaxenic clonal isolates originating from the Kattegat‐Skagerrak area (Godhe et al., 2013). Cultures were established as described, respectively. Strains represent two well‐characterized and genetically significantly differentiated local populations from distinct hydrographic areas/basins. From here on, estuarine Bothnian Sea strains are referred to as E1–E9 and the marine Kattegat‐Skagerrak strains are referred to as M1–M6, for each experiment (Table 1). All strains had previously been genotyped using eight polymorphic microsatellite markers (S.mar1‐8; Almany et al., 2009). The algal cultures were maintained in native seawater‐based silica enriched f/2‐medium (Guillard, 1975) with salinities resembling their native environment, 26 PSU (marine) or 7 PSU (estuarine). Experimental cultures were kept in 50‐ml culture flasks (Nunc EasYFlasks™ Nunclon™Δ) at 10°C, and the light–dark photo period was 12:12 h at a light intensity of 50 μmol photons m^−2^ s^−1^ (36 W Cool Daylight). Light and temperature conditions remained unchanged during all experiments.

**TABLE 1:** *S. marinoi* strain information

Strain	Strain designation	Location	Local sea surface salinity	Position	Collected (year)	Source
C1403	E1	Bothnian Sea	04‐06	N 62°07.15′, E 18°33.14′	2011	Sjöqvist et al. (2015)
C1406	E2	Bothnian Sea	04‐06	N 62°07.15′, E 18°33.14′	2011	Sjöqvist et al. (2015)
C1407	E3	Bothnian Sea	04‐06	N 62°07.15′, E 18°33.14′	2011	Sjöqvist et al. (2015)
C1412 (exp II)	E7	Bothnian Sea	04‐06	N 62°07.15′, E 18°33.14′	2011	Sjöqvist et al. (2015)
C1415 (exp II)	E8	Bothnian Sea	04‐06	N 62°07.15′, E 18°33.14′	2011	Sjöqvist et al. (2015)
C1416	E4	Bothnian Sea	04‐06	N 62°07.15′, E 18°33.14′	2011	Sjöqvist et al. (2015)
C1417	E5	Bothnian Sea	04‐06	N 62°07.15′, E 18°33.14′	2011	Sjöqvist et al. (2015)
C1425 (exp II)	E9	Bothnian Sea	04‐06	N 62°07.15′, E 18°33.14′	2011	Sjöqvist et al. (2015)
C1428	E6	Bothnian Sea	04‐06	N 62°07.15′, E 18°33.14′	2011	Sjöqvist et al. (2015)
V7	M1	Kattegat	25‐30	N 57°33.0′, E 11°31.5′	2009	Godhe et al. (2013)
V8	M2	Kattegat	25‐30	N 57°33.0′, E 11°31.5′	2009	Godhe et al. (2013)
V11	M3	Kattegat	25‐30	N 57°33.0′, E 11°31.5′	2009	Godhe et al. (2013)
St31	M4	Skagerrak	25‐30	N 58°51.0′, E 10°45.7′	2009	Godhe et al. (2013)
St51	M5	Skagerrak	25‐30	N 58°51.0′, E 10°45.7′	2009	Godhe et al. (2013)
Lys6S	M6	Skagerrak	25‐30	N 58°15.2′, E 11°03.5′	2009	Godhe et al. (2013)

### 2.2 Experiment 1: Reciprocal transplant experiments (estuarine vs. marine)

To test whether strains were locally adapted to home conditions, we carried out reciprocal transplant experiments whereby maximum growth rate was measured for strains growing in native and in non‐native water. Six estuarine strains (E1–E6) and six marine strains (M1–M6) were preacclimatized (as described below) to new conditions before maximum growth rate was measured in the non‐native treatment. Growth medium was prepared with natural seawater collected from the Skagerrak (marine water) and northern Baltic proper (estuarine) and double filtered through GF/F glass microfiber filter (pore size 0.7 μm; Whatman) and then Pall Supor Membrane filter (pore size 0.2 μm; Pall corporation). Preacclimatization was done in a stepwise manner by transferring estuarine strains from 100% Baltic Sea water to 75–50–25% (adding Skagerrak water) and finally 100% Skagerrak water, every 7 days. Marine strains were acclimatized using the same procedure but starting from 100% Skagerrak water and finishing in 100% Baltic Sea water. Preacclimatized strains were kept in new conditions for 7 days before measuring growth. The total duration of the acclimatisation procedure was 4 weeks, equivalent of 31 generations (7 generations per step, 1 generation = 1 mitotic cell division). Generally, at least six generations are recommended for acclimatization in microalgal work, while mutations and potential selections are expected to arise after 100 or more generations although no definite threshold has been determined (Zhang et al., 2021). Maximum growth rates were measured for each strain in triplicates. Growth was monitored daily by transferring 1 ml of each culture to a 48‐well plate and measuring chlorophyll fluorescence on a Thermo Scientific Varioskan® Flash (microplate reader) with SkanIt® Software 2.4.3 (Wavelength: Excitation (nm) 425, Emission (nm) 680). Maximum growth rate (*μ*_max_) was calculated as *μ*_max_ = (Ln *F*2 – Ln *F*1)/(*t*2 − *t*1), where *F*1 and *F*2 are the fluorescence at time points *t*1 and *t*2. Time points were selected based on the longest possible period of exponential growth, which was determined from growth curves established for each replicate experimental culture. Time points were taken at 3‐day intervals (Wood et al., 2005). Statistical test of fitness (maximum growth rates) differences was done using a mixed‐model two‐way ANOVA in IBM SPSS 27 for Mac. “Strain origin” (marine or estuarine) and “medium” (marine or estuarine seawater‐based) were defined as fixed factors, where “strains” was denoted as a random factor nested within strain origin. Residuals were tested for normality with the Kolmogorov–Smirnov test. In addition, overall means for each set of strains was calculated, and differences were tested statistically using a *t*‐test in Microsoft Excel 16.54 for Mac.

### 2.3 Experiment 2: Reciprocal transplant experiments with pH monitoring

In a second experiment, the same setup was used, with the specific aim to monitor pH, a potential abiotic factor to which populations can adapt. Experimental conditions were identical as above, except that strains E1, E4, and E5 (which had died) were replaced by strains E7, E8, and E9. In this second experiment, cell counts were estimated by measuring minimum fluorescence values (F0) using a Pulse Amplitude‐Modulation (PAM; WALZ®, MAXI version of IMAGING‐PAM *M‐Series*, model IMAG‐K4). The imaging‐PAM measurements of chlorophyll *a* fluorescence were done with a Kappa DX4‐285 (MAXI) camera. The parameter settings were as follows: intensity of the light 7, frequency 1, gain 3 and damping 2. The absorptivity of the Red Gain was 40, Red Intensity 4 and NIR Intensity 3. The intensity of the Saturation Pulse was 8. To standardize cell counts, dilutions were made to achieve 100%, 50%, 10%, 5%, 1% and 0.1% of medium concentration in a 48‐well plate. The 48‐well plate was placed in the dark for 15 min, to allow cultures to acclimatize before measuring. Cell density was monitored at the same time daily, 11 days in a row. After the fluorescence/density was measured with the PAM, a subsample (1 ml) from of each bottle was fixed with Lugol's solution in a 48‐well plate. The pH of all the 72 bottles was measured at 3‐day intervals using the HI 2221 Calibration check pH/ORP Meter of Hanna Instruments (pH electrode HI 1131P, temperature probe HI 7662; accuracy: ±0.01 pH and ±0.2°C). This was calibrated with buffers of 4.01, 7.01 and 10.01 pH. During calibration, the pH value was automatically calibrated to the corresponding temperature as this was measured too. Statistical analyses were performed as above. Moreover, cell density was plotted against pH to establish the correlation coefficient.

### 2.4 Experiment 3: Competition experiment

After acclimatization, we had two batches of each strain from Experiment 1: one batch which had remained in its native water (*adapted* to estuarine or marine water), and the other in the new non‐native water (*acclimatized* to estuarine or marine water). Using a common garden setup, we inoculated an *adapted* strain together with an *acclimatized* strain to the marine Skagerrak water and estuarine Baltic Sea water. In total, there were seven different two‐strain combinations of an estuarine and a marine strain: M1 and E3 (P1), M3 and E3 (P2), M3 and E4 (P3), M3 and E5 (P4), M5 and E3 (P5), M5 and E4 (P6) and M5 and E5 (P7). Only a subset of the strains used in Experiment 1 could be included in Experiment 3, because the microsatellite allele‐specific quantitative PCR technique (see details in 2.6) was not unbiased in all strain combinations. The strain composition at the end of the experiment was determined using a microsatellite allele‐specific quantitative PCR technique (Sefbom et al., 2015; see details in 2.6). Strain combinations had been chosen so that the relative abundance of each strain in combination with another could reliably be quantified based on fragment peak‐heights in an electropherogram. Each of the seven strain combinations, in the two treatments, was grown in triplicates. Both strains were inoculated at the same time at equal concentrations of 5000 cells per ml (total starting concentration of 10,000 cells per ml). Growth was monitored daily using fluorescence as described for Experiment I. The experiments were terminated in the early stationary phase (between day 8 and 10 depending on strain combination). On the final day of the experiments, each replicate was filtered onto 3.0‐μm filters (Ø25 mm Versapore®‐3000, Pall Corporation) and kept at −80°C until DNA extraction.

### 2.5 DNA extraction and microsatellite analysis

Genomic DNA was extracted using a cetyltrimethylammonium bromide protocol based on Kooistra et al. (2003). Four microsatellite loci were amplified, S.mar1, S.mar 4, S.mar5 and S.mar6 (Almany et al., 2009), with PCR conditions as described by Godhe and Härnström (2010). The products were analysed in an ABI 3730 (Applied Biosystems) and allele sizes were assigned relative to the internal standard GS600LIZ. Binning and peak‐height determination was performed using GeneMapper (ABI Prism®GeneMapper™Software Version 3.0).

### 2.6 Allele‐specific quantitative PCR (AsQ‐PCR)

To assess relative abundance of the experimental mixed strain cultures, we used an AsQ‐PCR method described by Meyer et al. (2006) and optimized for *S. marinoi* in Sefbom et al. (2015). The respective peak‐heights from the two strains in the electropherograms are used as a relative quantification measurement. To establish that PCR amplification did not favour one strain over the other, we mixed the seven strain combinations in five known proportions (based on microscopic cell counts), ranging from 10:90 to 90:10 (3 replicates each), and carried out DNA extraction and microsatellite analysis as described above. Four different microsatellite markers were tested (S.mar1, S.mar 4, S.mar5 and S.mar6) in order to find the least biased PCR. Peak‐height relative abundances were plotted against known relative abundances to obtain *r* ^2^‐values. Strain combinations with peak‐height ratios representative of the mixed cell ratios (*r* ^2^ > 0.9) were considered as having unbiased PCRs and reliable for assessing competition experiments. To determine whether the relative proportion of a strain was significantly higher when competing in its native environment we utilized a one‐way randomized block ANOVA in IBM SPSS 27. “Growth medium” (marine or estuarine) was used as a fixed factor, and “strain combinations” were designated as random factors. The proportion of marine strains was used as the response variable (not both, since the proportion of one strain is always one minus the other strain). Residuals were tested for normality.

## 3 RESULTS

### 3.1 Reciprocal transplant experiments (experiment 1 and 2)

Single strain growth in native and non‐native conditions was measured over a period of 13–15 days for Experiment 1 (Figure S1). When all the strains were grown in their respective native conditions, they entered stationary phase after 9–10 days. After 13 days, the native salinity treatment was ended. When strains were grown in non‐native conditions, the estuarine strains showed no indications of a lag phase and entered the stationary phase after 9–10 days, whereas marine strains grew slower and had not entered stationary phase by the end of the experiment. After 15 days, the non‐native salinity treatment was ended. Cell densities at the termination of the experiment were calculated (based on fluorescence measures) and varied between 1.3 and 2.0 × 10^5^ cells per ml.

**FIGURE 1:**
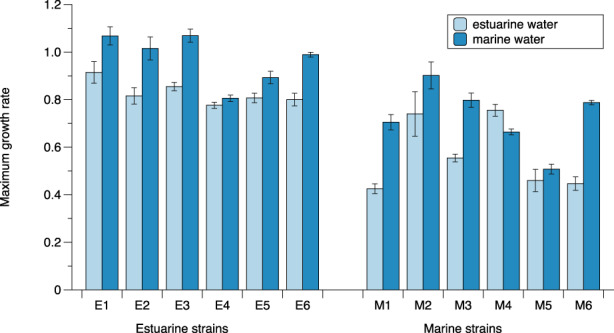
(a) Average maximum growth rate (calculated from fluorescence measurements, *N* = 3) per strain cultured in estuarine water (salinity 7 PSU) and marine water (salinity 26 PSU) conditions. E1–E6 refer to estuarine strains and M1–M6 to marine strains. Error bars indicate standard deviation of the mean (*N* = 3)

Marine strains performed better in native than non‐native conditions, whereas estuarine strains performed better in non‐native conditions (Figure 1). Both marine and estuarine strains displayed higher maximum growth (*μ*_max_) rates when grown in marine water (Figure 1). Growth rate was significantly affected by both strain origin (ANOVA, *F* = 20.385, *p* = 0.001) and medium (*F* = 85.131, *p* < 0.001), but not by the interaction between origin and medium. When the mean was calculated for the whole population (six strains each), the mean growth rate in the marine water was 0.97 for the estuarine strains and 0.72 for the marine strains (*t*‐test *p* = 0.006). Likewise, in the estuarine treatment, the overall mean for estuarine strains was 0.83 but only 0.56 for the marine stains (*t*‐test, *p* = 0.006).

**FIGURE 2:**
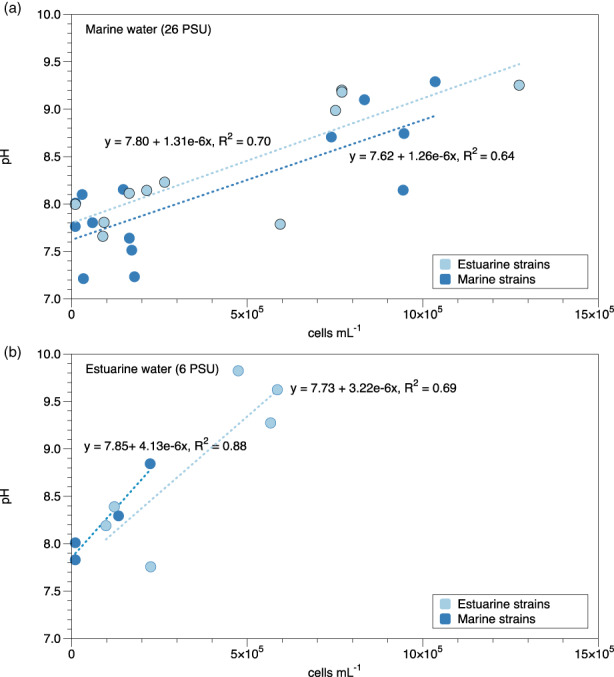
(a) Measured pH level at different mean cell concentrations of estuarine (light blue) and marine (dark blue) strains grown in marine water. (b) Measured pH level at different mean cell concentrations of estuarine (light blue) and marine (dark blue) strains grown in estuarine water. Dotted lines show linear correlation, equation provided with *R* ^2^ value

The repeated transplant experiment (2) in which pH was also measured showed the same pattern as the first reciprocal transplant experiment (Figure S2). As expected, pH increased with increasing cell densities in both treatments. This relationship was highly correlated, with *R* ^2^ values ranging from 0.63 to 0.88 (Figure 2). In marine water, the final pH and cell numbers were similar for estuarine and marine strains. However, in low‐salinity estuarine water, marine strains stopped growing at a pH between 8.5 and 9 when cell concentrations were around 2.5 × 10^5^ cells per ml. In contrast, estuarine strains continued growing to a pH above 9.7 representing cell concentration of 4–6 × 10^5^ cells per ml (Figure 2).

### 3.2 Competition experiments

**FIGURE 3:**
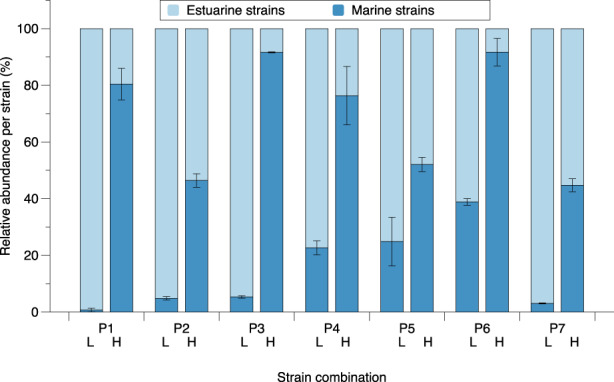
Average relative abundance of estuarine strain versus marine strain after 8–10 days (depending on when strain pairs reached early stationary phase), growing together in marine high salinity (H) and estuarine low (L) salinity F/2 medium. Relative abundances were established using AsQ‐PCR. P1–P7 refer to specific strain combinations. Error bars indicate standard deviation of the mean (*n* = 3)

The standard curves plotted between electropherogram peak‐height ratios and known mixed cell abundances resulted in *r* ^2^‐values of 0.93–0.99 (Figure S3). For the four loci tested (S.mar 1, S.mar 4, S.mar 5 and S.mar 6), different loci showed least bias depending on the strain combination. The least biased locus was therefore used to assess the relative abundance of each strain in the respective strain pairs. In all seven combinations, strains performed better in their respective native conditions (Figure 3), that is, when strains grew in native salinity conditions, they significantly increased their final relative abundance (ANOVA; *F* = 226.030; *p* < 0.001) compared to when they grew in non‐native conditions (Figure 3).

## 4 DISCUSSION

Within microalgae, numerous studies have shown high genetic differentiation among populations despite potentially high dispersal potential (Adams et al., 2009; Nagai et al., 2007; Rengefors et al., 2012). Although so far there has been little focus on the drivers of differentiation in microalgae, a few studies have demonstrated ecological differentiation (Postel et al., 2020; Rynearson & Armbrust, 2004; Sjöqvist et al., 2015; Škaloud & Rindi, 2013; Weisse et al., 2011). Here, we hypothesized that local adaptation to different seawater chemistry provides a barrier to gene flow between estuarine (Bothnian Sea) and marine populations (Skagerrak/Kattegat) of the microalga *S. marinoi*. These sites are located far apart, but directional migration has been detected, indicating gene flow (Sjöqvist et al., 2015) even though currents are expected to act as dispersal barriers. While competition and transplant experiments provided evidence that the marine strains were locally adapted, the picture was less clear for the estuarine strains. The latter grew best in the more saline marine water but were nevertheless outcompeted in four out of seven pairs by marine strains, when co‐grown in marine conditions. These results, and implications for mechanisms involved in population differentiation in microorganisms, are discussed below (Figure 3).

When testing for local adaptation in the transplant experiments, we expected that local genotypes would exhibit higher growth rates than foreign genotypes when grown in the same salinity conditions. However, this was not entirely the case. While marine strains grew less well in estuarine water than estuarine strains, strains from both populations displayed highest growth rate in high‐salinity seawater medium. Moreover, estuarine strains had significantly higher maximum growth rates, compared to marine genotypes in both water types. The higher fitness observed in estuarine strains when grown in marine water could have two possible explanations: (i) a confounding effect of intrinsic water quality (Kawecki & Ebert, 2004), that is, for reasons other than salinity the marine water provided a better environment for growth, alternatively (ii) this is a product of countergradient selection resulting in countergradient variation (CnGv) in estuarine strains (Conover & Schultz, 1995; Levins, 1969). Even if the former explanation is correct, and marine water served as better substrate for growth, it does not explain why estuarine strains grew better than the native marine strains in both conditions. CnGV, on the other hand, could explain this effect. CnGV is characterized by the opposition of environmental and genetic effects (Conover & Schultz, 1995). This means that the estuarine strains have adapted to the comparatively challenging environment of the estuarine Baltic Sea by increased intrinsic growth rates to compensate for the poor conditions in estuarine waters. As a result, they grow even faster than marine strains when transferred to the more favourable marine environment. In contrast, the marine strains have not adapted to the estuarine conditions, and their growth there is reduced due to suboptimal growth conditions, compared to the native estuarine strains.

We postulated that not only salinity but also tolerance to pH could play a role in local adaptation and therefore added the experiment in which we monitored pH. Different phytoplankton species have different tolerance to pH, and pH ≥9 is lethal to many species (Goldman et al., 1982; Hansen, 2002). Diatoms in coastal areas are often sensitive to elevated pH (above pH 8.7–9.1; Lundholm et al., 2004), while some bloom‐forming species tolerate pH above 9.0 (Hansen, 2002; Hansen et al., 2007). For *S. marinoi*, Andersson et al. (2020) observed growth limitation already at pH 8.5. Cell concentration (phytoplankton biomass) and pH are tightly coupled since photosynthesis directly affects ambient pH. During carbon (CO_2_ and HCO_3_^−^) uptake in photosynthesis, [H^+^] ions are removed, thereby increasing pH in the water. The extent of the pH increase depends on the buffering capacity (alkalinity) of the ambient water, and water with high alkalinity has less variation in pH than water with low alkalinity. Relevant in the current case is that marine water has a higher buffering capacity than the estuarine water in the Bothnian bay of the Baltic Sea (Key et al., 2004; Thomas & Schneider, 1999). As a result, surface water in the marine Skagerrak/Kattegat is usually around ~7.9–8.2 while pH in the Baltic Sea (e.g. Gulf of Finland) ranges from 7.4 to 9.2 (Brutemark et al., 2011). Hence, it is plausible that cells in an environment with larger fluctuations in pH will evolve higher tolerance to pH changes. Monitoring of pH in Experiment 2 provided some support of this hypothesis. While pH was the same at the beginning of the experiments, the endpoints differed. Estuarine strains were able to tolerate higher pH and thereby reach higher cell numbers than the marine strains when grown in low‐salinity water.

Differences in pH tolerance between marine and estuarine strains can thus explain why marine strains do less well in estuarine water as the cultures grew dense. In the estuarine treatments of the competition experiment, with water of lower buffer capacity, pH presumably increased beyond 8.5, thereby likely inhibiting the marine strains while estuarine strains could maintain growth. The cell densities in these experiments were mostly much above 1.5 cells per ml, the point at which cells switch from exponential to linear (limited growth rates; Andersson et al., 2020).

Interestingly the competition experiments show a competitive advantage of local populations in their home environment, which in the case of the marine environment suggests a cost involved in the higher growth rate of the estuarine strains. This cost seems to be connected to the estuarine strains' competitive ability. That is, estuarine genotypes were unable to dominate when competing with the marine genotypes in marine water. This indicates that fitness measures determined in monoculture, such as growth rate, cannot alone explain the competitive abilities and that for the estuarine strains, there is a trade‐off between traits that favour growth rate and traits that favour competition. Contradictory results between monoculture fitness and competitive ability have been shown previously (e.g. Collins & Schaum, 2021; Roger et al., 2012; Sefbom et al., 2015). Collins and Schaum (2021) demonstrated that microalgal lineages can detect non‐self‐conspecifics (other strains than self) and modulate their growth rate even if their abiotic environment has not changed. When co‐grown, all lineages increased growth rate (although to different degrees), which was attributed to a re‐allocation of carbon from net photosynthesis to growth (rather than for example storage; Schaum & Collins, 2014). Thus, it is possible that in our experiments, growth rate or growth curve in the estuarine strains changed, when co‐grown with marine strains in marine water.

Ultimately, we provide evidence for local adaptation and suggest that the mechanism includes countergradient selection. We show that adapted traits in local genotypes provide a competitive advantage that results in an increased abundance compared to nonlocals. This competitive advantage implies that despite dispersal potential mediated by oceanographic connectivity, not least from inside the Baltic Sea to the outside, the presence of locally adapted native populations may prevent establishment of immigrant strains. In this way, population divergence is further reinforced beyond differences caused by isolation and genetic drift (Boileau et al., 1992). In fact, local adaptation may provide the most important mechanism that reduces gene flow and causes population genetic differentiation in a species, such as *S. marinoi*, in which differentiation between geographically close populations is established in the absence of physical barriers.

## ACKNOWLEDEGMENTS

Funding for this work was provided by a grant of the Swedish Research Council FORMAS to KR and AG (215‐2010‐751), Academy of Finland (Decision No. 310449) to AK. We thank Robin Petersson and Nuria Simoens for help with laboratory experiments. Dr. Anders Nilsson is thanked for assistance with statistical analyses.

## CONFLICT OF INTEREST

There is no conflict of interest for this work.

## Supporting information


Figure S1

Figure S2

Figure S3

## Data Availability

The data that support the findings of this study are openly available in Dryad Digital Repository at https://doi.org/10.5061/dryad.xpnvx0kh7.
