# Supplementary material for: Local adaptation through countergradient selection in northern populations of Skeletonema marinoi
Source: Evol Appl. 2022 Jul 11;16(2):311–20. doi: 10.1111/eva.13436 (PMC9923485; doi:10.1111/eva.13436)
Supplement: Supplementary file 1 — Figure S1 [file EVA-16-311-s001.docx]

**Sefbom et al. Supplementary Material**

**Supplementary Figure 1**

Suppl Fig. 1. Averaged growth curves for Baltic Sea and Skagerrak strains growing in a) native water, b) non-native water.
