# Supplementary material for: Local adaptation through countergradient selection in northern populations of Skeletonema marinoi
Source: Evol Appl. 2022 Jul 11;16(2):311–20. doi: 10.1111/eva.13436 (PMC9923485; doi:10.1111/eva.13436)
Supplement: Supplementary file 2 — Figure S2 [file EVA-16-311-s002.docx]

Sefbom et al. Supplemental Figure 3

A)

Supplemental figure 3. Average maximum growth rate (measured as fluorescence) per strain cultured in estuarine water (salinity 7 PSU) and marine water (salinity 26 PSU) conditions. E1, E2, E7, E8, E9, E6 refers to specific estuarine strains. M1-M6 refers to specific marine strains. Error bars indicate standard deviation of the mean, n=3.
