# Supplementary material for: Local adaptation through countergradient selection in northern populations of Skeletonema marinoi
Source: Evol Appl. 2022 Jul 11;16(2):311–20. doi: 10.1111/eva.13436 (PMC9923485; doi:10.1111/eva.13436)
Supplement: Supplementary file 3 — Figure S3 [file EVA-16-311-s003.docx]

**Figure S 3a-g**. The relative abundance of one strain, in a two-strain mix, determined by microscope cell count on the x-axis, and proportional peak heights of the respective strains in the electropherogram after fragment amplification on the y-axis. a) strains V7 and C1407; b) strain V11 C1407; c) strain V11 and C1416; d) strains V11 and C1417; e) strains St51 and C1407; f) St51 and C1416; g) strains St51 and C1417.
